# Navigating care: The burden on caregivers of children with hydrocephalus in South Africa’s teaching hospitals

**DOI:** 10.4102/hsag.v31i0.3580

**Published:** 2026-10-30

**Authors:** Makwena M. Sibuyi, Yvone L. Molomo, Nthati L. Matebula, Thembelihle S. Ntuli

**Affiliations:** 1 Department of Physiotherapy, School of Health Care Sciences, Sefako Makgatho Health Sciences University, Tshwane, South Africa; 2 Department of Physiotherapy, Polokwane Hospital, Polokwane, South Africa; 3 Department of Physiotherapy, Dr. George Mukhari Academic Hospital, Tshwane, South Africa; 4 Department of Statistical Sciences, School of Science and Technology, Sefako Makgatho Health Sciences University, Tshwane, South Africa

**Keywords:** caregiver burden, hydrocephalus, paediatric patients, South Africa, academic hospitals

## Abstract

**Background:**

Caregivers of children with hydrocephalus in low-resource settings face profound challenges, yet their experiences within the South African context are poorly documented.

**Aim:**

This study aimed to investigate the interrelated challenges encountered by caregivers of paediatric patients with hydrocephalus.

**Setting:**

This study was conducted at two academic tertiary hospitals in Limpopo and Gauteng provinces.

**Methods:**

A qualitative exploratory study using semi-structured interviews recruited 39 caregivers of paediatric patients with hydrocephalus. Data were analysed using inductive thematic analysis in NVivo 16 software, and the analysis was halted at saturation.

**Results:**

Caregivers were predominantly young and unemployed. Four overarching thematic challenges emerged: (1) health system challenges; (2) socioeconomic consequences of caring for a child with hydrocephalus; (3) psychosocial and caregiving burden; and (4) formal social protection availability.

**Conclusion:**

Caregivers endure severe, interconnected systemic, financial, and psychosocial burdens that critically compromise their well-being and care capacity. Findings underscore an urgent need for holistic, family-centred interventions, including systemic healthcare reforms, economic protection, integrated psychosocial support, and structured caregiver education programmes to mitigate this crisis and improve patient outcomes.

**Contribution:**

The study advances the understanding of how health system limitations intersect with poverty, amplifying caregiver vulnerability and inequity.

## Introduction

Hydrocephalus, characterised by an abnormal accumulation of cerebrospinal fluid within the brain, is a major cause of neurological morbidity among children globally. The burden of the condition is disproportionately higher in low- and middle-income countries (LMICs), where incidence is often elevated because of preventable causes, mostly post-infectious complications (Dewan et al. 2018). The standard treatment involves surgical diversion of cerebrospinal fluid, most commonly through ventriculoperitoneal (VP) shunt insertion. Although palliative and sometimes lifesaving, this intervention is associated with high rates of complications, including infection, obstruction, and mechanical failure, often necessitating repeated hospitalisations and long-term clinical follow-up (Chaichana & Cutsforth-Gregory 2025).

In sub-Saharan Africa, and particularly in South Africa (SA), the management of paediatric hydrocephalus is further complicated by health system constraints, resource limitations, and pronounced socioeconomic inequalities (Enslin et al. 2021; Kantawala et al. 2023). Specialised neurosurgical services are largely centralised within tertiary and academic hospitals, which serve as referral centres for complex paediatric conditions. While these facilities play a critical role in acute management, the long-term care of children with hydrocephalus extends beyond the hospital setting and relies heavily on family caregivers. These caregivers are responsible for ongoing monitoring of signs and symptoms of shunt complications, adherence to follow-up care, and early identification of symptoms that may alert to complications, making them central to successful disease management (Geuze & Goossensen 2019; Torres & Ohajunwa 2025).

Caregiver burden is increasingly conceptualised as a multidimensional construct encompassing psychological, physical, social, and financial domains, particularly in the context of chronic paediatric conditions. Within this framework, caregiving is not a singular responsibility, but a complex and sustained activity shaped by both individual and structural factors. Existing literature indicates that caregivers of children with hydrocephalus experience significant emotional distress and financial strain because of direct and indirect costs, and considerable physical demands, particularly when children present with associated disabilities or developmental delays (Knerlich-Lukoschus et al. 2024; Mwiinga, Malekani & Mwape 2023; Shahali et al. 2024). In LMIC settings, these challenges are often intensified by limited access to healthcare services, inadequate social support systems, and broader structural inequities.

In the South African context, additional factors such as geographic barriers to accessing centralised specialist care, cultural perceptions of disability, and the strain on an overburdened public health system further compound caregiver burden (Trani et al. 2020). Despite the critical role caregivers play in influencing health outcomes, there remains a paucity of context-specific research focusing on caregivers of children with hydrocephalus in SA. Much of the existing literature is derived from high-income settings or other regions of sub-Saharan Africa and may not adequately capture the unique intersection of socioeconomic vulnerability and health system constraints present in SA. Furthermore, limited attention has been given to how structural, financial, psychosocial, and health system factors interact to shape caregiving experiences in this setting.

This study therefore aims to explore the challenges experienced by caregivers of paediatric patients with hydrocephalus receiving care at South African academic tertiary hospitals. By examining caregiving experiences across medical, financial, psychosocial, and health systems, this study seeks to generate contextually relevant evidence to inform our understanding of these experiences. From this information, the study may suggest targeted interventions, strengthen caregiver support systems, and contribute to more holistic and responsive models of care. Ultimately, understanding these experiences is essential to improving both caregiver wellbeing and child health outcomes in resource-constrained settings.

## Research methods and design

The interpretivist theory was deployed. Interpretivism assumes that reality is subjective, multiple, and socially constructed (Kumatongo & Muzata 2021). The use of interpretivism allows for multiple explorations of the various dimensions of the truth.

### Study design

A qualitative exploratory study using semi-structured interviews with caregivers of paediatric patients with hydrocephalus was employed. This design seeks to provide a rich and in-depth experiences for participants while remaining close to their own perspectives and interpretations (Abu-Halaweh et al. 2021).

### Study population and setting

The semi-structured interviews were conducted in the specialised paediatric neurosurgical wards of two tertiary academic hospitals in SA: Polokwane Provincial Hospital in the Limpopo province and Dr George Mukhari Academic Hospital in Gauteng province. These academic hospitals are centralised to provide specialised medical services to lower-hospital settings, such as district and regional hospitals (Fusheini, Eyles & Goudge 2017). In these hospitals, paediatric patients are managed by multidisciplinary teams at academic hospitals, including paediatric neurosurgeons, paediatricians, and other healthcare professionals.

### Sampling technique and sample size

A total population sampling approach (Etikan, Musa & Alkassim 2016) was used to recruit parents or caregivers of paediatric patients diagnosed with hydrocephalus, resulting in a final study sample of 39 participants. This approach was considered appropriate because the study was conducted in tertiary teaching hospitals that provide specialised neurosurgical services for children with hydrocephalus. As hydrocephalus is a relatively specialised condition managed at these centres, the number of eligible caregivers attending the hospitals during the data collection period was limited. Thus, only caregivers whose child was hospitalised were interviewed.

### Inclusion criteria

All caregivers who were with their hospitalised child at the tertiary academic hospital during the study period were invited to participate in the study.

### Data collection

Data were collected through individual semi-structured interviews conducted by selected physiotherapists working in the paediatric wards of the chosen tertiary academic hospitals. As data collectors, physiotherapists signed a non-disclosure agreement prior to the commencement of data collection. Potential caregivers were identified from ward admissions and were approached and invited to participate. The study information was written and explained to participants, and they consequently signed written informed consent. Caregivers were given the opportunity to select a date and time that was convenient for them.

The data collectors were briefed by the principal investigator on the study objectives and the use of the interview guide to ensure consistency in the data collection procedure. An interview guide consisted of closed and open-ended questions. Closed-ended questions were used to collect demographic information, including the caregiver’s and the child’s age, marital status, employment status and educational level. Open-ended questions explored caregivers’ experiences of caring for a child with hydrocephalus, and follow-up probing questions were used to elicit further detail and clarify caregivers’ responses. To facilitate reliable data collection and participation, interviews were conducted in the language preferred and best understood by the caregivers. Interviews conducted in languages other than English were translated into English, then transcribed verbatim. Interviews were conducted in a private room within the hospital to ensure confidentiality and minimise interruptions from the ward environment. With participants’ permission, interviews were audio-recorded and lasted approximately 40 min. Each transcript was allocated a unique identity number for the sake of confidentiality (Kaiser 2009).

Data collection took place over 9 months from April 2025 to December 2025.

### Data analysis

All interview transcripts were uploaded into NVivo qualitative data analysis software version 16 (Lumivero, Denver, Colorado, United States) (Lumivero 2025) to facilitate data management and analysis. Data were analysed by the authors (MMS, TSN) using a six-stage inductive thematic analysis approach (Dawadi 2021). Thematic analysis followed an iterative process of data familiarisation, coding, category development, and theme generation (Dawadi 2021). Initial codes were derived from topics identified in the narratives of the study’s participants and subsequently grouped into categories, which were then refined into overarching themes through discussion and consensus among the authors. Representative verbatim quotations were selected to illustrate each theme and subtheme and reflect participants’ experiences. Data saturation was reached when new data became a repeat of previous data and participants did not have differing views or experiences (Francis et al. 2010).

### Trustworthiness

Trustworthiness of the study was ensured by adhering to five strategies: Credibility, reflexivity, dependability, confirmability, and transferability (Johnson, Adkins & Chauvin 2020). Credibility was upheld through audio-recorded interviews and prolonged engagement with participants that ensured accurate data collection. In demonstrating reflexivity, the data collectors were familiar with the hospital environment and had extensive experience in working with caregivers. Although their experience added value in interviewing caregivers, it is acknowledged that their roles could have influenced data collection. In addition, caregivers may have perceived the data collectors as members of the healthcare team, potentially affecting their willingness to disclose negative experiences or criticisms of the healthcare system. To minimise these influences, participants were informed that participation was voluntary, that their decision to participate or decline would not affect the care their child received, and that all responses would remain confidential. To ensure transferability, descriptions of the province name, the district names, participants’ demographics, data collection procedures, and duration were provided in the data collection (Johnson et al. 2020). The findings are dependable if the same interview guide, participants and similar context are used for another study. Lastly, confirmability was upheld through collaborative efforts with a co-coder, ensuring consensus and resolving any coding discrepancies that arose during the process (Nowell, Norris, White et al. 2017).

### Ethical considerations

Ethical approval for this study was obtained from the Research Ethics Committee of Sefako Makgatho Health Sciences University (Reference Number: SMUREC/H/375/2024:IR), the Provincial Department of Health in Limpopo (Ref: LP 202504018) and Dr George Mukhari Academic Hospital. The research was conducted in strict compliance with the ethical principles for medical research involving human subjects as outlined in the Declaration of Helsinki (World Medical Association 2013). Written informed consent was obtained from each caregiver who chose to take part, with the understanding that participation was entirely voluntary and that they retained the right to withdraw at any time without affecting their child’s medical care.

Given the potentially sensitive nature of discussing caregiving experiences and emotional challenges associated with caring for a child with hydrocephalus, arrangements were made with a psychologist to provide support should any participant experience concerns requiring further assessment or professional support, including emotional distress, and be referred to the appropriate healthcare professional. All personally identifiable information was removed to maintain confidentiality (Brennan 2022).

## Results

### Demographic characteristics

The study included 39 caregivers of children with hydrocephalus, as illustrated in Table 1. The median age was 30 years (interquaterile range [IQR] 12), and all were female. Educational attainment was generally low, with 22.5% (*n* = 9) having tertiary education and 60.0% (*n* = 24) having completed only secondary school. Unemployment affected 95.0% (*n* = 38) of caregivers.

**TABLE 1:** Demographic characteristics of caregivers’ (*N* = 39).

Characteristics	Variable	*n*	%
Caregivers’ age (years)	< 20	2	5.0
	20–29	16	40.0
	30–39	14	35.9
	> 40	7	17.5
Children’s age	< 6 months	10	25.6
	6–11 months	12	30.8
	1–2 years	11	28.2
	3–5 years	3	7.7
	≥ 6 years	3	7.7
Caregivers’ education level	Primary	7	17.5
	Secondary	24	60.0
	Tertiary	8	20.5
Caregivers’ employment status	Unemployed	37	94.9
	Employed	2	5.0

### Challenges of caregiving

An inductive thematic analysis of caregiver experiences reveals a range of healthcare system challenges, which have been organised into three overarching themes and sub-themes, as summarised in Table 2.

**TABLE 2:** Themes and sub-themes.

Themes	Sub-themes
1. Health system challenges	1.1 Shunt problems and recurrent hospitalisation 1.2 Appointment delays and prolonged waiting times 1.3 Education and counselling by health care workers
2. Socioeconomic consequences of caring for a child with hydrocephalus	2.1 Transport problems 2.2 Loss of income and employment opportunities
3. Psychosocial and caregiving burden	3.1 Psychological and emotional distress 3.2 Family disruption and neglect 3.3 Relentless caregiving responsibility 3.4 Social isolation and stigma
4. Formal social protection availability	4.1 Reliance on informal support networks 4.2 Insufficient formal support 4.3 Reluctance to access psycho-social services

### Theme 1: Health system challenges

Caregivers’ experiences highlighted structural barriers within the health system that not only limited access to care but also imposed high socioeconomic costs. Shunt-related complications requiring recurrent hospitalisation, gaps in caregiver education, and the need for frequent travel to centralised facilities resulted in financial strain and loss of income opportunities for caregivers. These issues are interconnected and show how the health system places substantial responsibility for long-term care on caregivers, thereby exacerbating the burden on already vulnerable households.

#### Sub-theme 1.1: Shunt problems and recurrent hospitalisation

This subtheme describes the serious health consequences that result directly from shunt failures. The need for numerous repeat surgeries, the onset or worsening of severe symptoms (such as vomiting and crippling headaches), the regression of a child’s developmental progress, and the increased risk of hospital-acquired infections are the negative consequences that are set off by these critical events. Participants described a reality of being constantly ‘in and out of hospital’, adhering to frequent outpatient check-ups, and navigating stressful referrals between different treatment facilities.

This was supported by a participant who describes a relentless and exhausting cycle driven by medical complications:

‘Recurrent admissions to hospital, having to come back to hospital many times, shunt malfunction and multiple operations.’ (P2, 28 years, female, unemployed)

One of the participants also describes a cycle where access barriers directly lead to acute medical crises and repeated operations by saying:

‘Our hospital is too far to reach. My baby fitted for a long time whilst waiting for ambulance to arrive. This caused complications to my baby. The shunt malfunctioned many times and led to recurrent hospital admissions.’ (P7, 37 years, female, unemployed)

#### Sub-theme 1.2: Appointment delays and prolonged waiting times

A core challenge within the healthcare system was the pattern of prolonged hospital inpatient care and often waiting for a long time before treatment is initiated. These delays have caused frustration for caregivers navigating logistical bottlenecks, limited healthcare resources, and complex referral pathways. This has led to an erosion of trust in the healthcare system and yielded concerns that the delay itself could adversely affect the prognosis of the condition in their child with hydrocephalus. This was outlined by a participant who shares a story of initial success followed by a confusing and frightening setback, compounded by a serious new condition and systemic delays:

‘My child been in hospital for three weeks, and nothing has been done, no medication … watching your child wait in bed while the disease doesn’t wait.’ (P16, 23 years, female, unemployed)

Another participant said:

‘Since the baby was born, I never left the hospital. I’m trapped here, waiting for the next step, while my life outside falls apart. My maternity leave will end, and I might lose my job. The delay in her care isn’t just about her health; it’s destroying our entire future.’ (P14, 26 years, female, unemployed)

Another participant explains how a single prolonged hospitalisation creates cascading problems for the entire family:

‘Staying in the hospital for a long time. This child is consuming the money for other children because she is not receiving grant. I am always in the hospital.’ (P6, 43 years, female, unemployed)

#### Sub-theme 1.3: Education and counselling by healthcare workers

Participants’ experiences with caregiver education were inconsistent among themselves. Some reported receiving adequate information about the child’s condition and its management, which supported their preparedness.

This is supported by a participant who said:

‘I am coping with good support. They gave me education and counselling about what can go wrong and right with a shunt. Knowing what to look for, what the signs mean, it doesn’t take the fear away, but it gives me power. I feel like I am ready for anything because I am not in the dark.’ (P13, 25 years, female, unemployed)

Others indicated that they received little to no information, leading to confusion, fear and difficulty accepting the child’s condition. As a participant expressed the terror of the unknown:

‘This is new to me, and I don’t understand. I am very concerned. Every new symptom is a terrifying mystery … the not-knowing is a constant torture.’ (P12, 24 years, female, unemployed)

Another participant expressed similar sentiments:

‘I recently found out. I am still shocked, trying to accept the situation … the ground is still shaking under my feet. The words they use, the future they describe … it feels like they’re talking about someone else’s life.’ (P1, 26 years, female, unemployed)

Another participant described a life circumscribed by fear:

‘I am always afraid. Afraid my second baby will also have this condition … My life is ruled by “what if.” I can’t plan; I can’t relax.’ (P4, 45 years, female, unemployed)

### Theme 2: Socioeconomic consequences of caring for a child with hydrocephalus

#### Sub-theme 2.1: Transport problems

The largest immediate financial hardship was found to be the ongoing, unavoidable transport expenses required to obtain healthcare. Participants frequently emphasised how transport costs strain tight household budgets and must be covered by social subsidies intended for basic sustenance. As a result, access to healthcare becomes increasingly dependent on the availability of social support, compelling caregivers to make difficult trade-offs between their child’s healthcare needs and their family’s daily survival. This was expressed by a participant who said:

‘The transport cost is a monster … The South African Social Security Agency [*SASSA*] grant … gets eaten by taxi fares.’ (P6, 23 years, female, unemployed)

With harsh clarity, another participant reframed the health system failure:

‘The grant isn’t support; it’s a travel voucher for a journey we never wanted to take.’ (P15, 39 years, female, unemployed)

#### Sub-theme 2.2: Loss of income and employment opportunities

Participants reported job loss, reduced work hours, and abandoned education as direct consequences, sacrificing not just present income but also future earning capacity. This creates a permanent setback, locking families into poverty. The testimonies of Participant 10 and Participant 9 powerfully express this sacrifice:

‘How can I find a job? I have to go to the hospital every single month. No employer will accept that. The money to go up and down makes it impossible to work, and not working makes it impossible to have money. It’s a trap with no exit.’ (P10, 28 years, female, unemployed)

Meanwhile, the other participant articulates the direct theft of human possibilities by saying:

‘I’m missing time for my practicals at school. My education, my chance to build a better future, is being sacrificed because I have to stay at the hospital for long periods. My child’s illness is stealing my own potential to earn.’ (P9, 45 years, female, unemployed)

### Theme 3: Psychosocial and caregiving burden

Caregivers described a substantial psychosocial burden arising from the ongoing demands of caring for a child with hydrocephalus. This burden was characterised by persistent psychological and emotional distress, compounded by the relentless nature of caregiving responsibilities. In addition, caregivers reported significant disruptions to family dynamics, including neglect of other children, as well as experiences of social isolation and stigma, which reflected the broader relational impact of caregiving.

#### Sub-theme 3.1: Psychological and emotional distress

This subtheme describes the substantial and diverse psychological burden that carers experience, extending beyond transient worry to encompass clinically significant emotional states. One caregiver expressed the overwhelming emotional burden, explaining that:

‘I am not emotionally okay … the constant worry about the child creates a weight that just crushes your spirit. You feel broken in a way sleep can’t fix.’ (P9, 45 years, female, unemployed)

Another participant described the unrelenting nature of caregiving as inherently depressing:

‘Always looking after the child can be depressing. There is no break, no moment when you are not “on.”’ (P11, 40 years, female, unemployed)

#### Sub-theme 3.2: Family disruption and neglect

The findings reveal the collateral impact on family dynamics, highlighting how the caregiving crisis disrupts the entire family system. This includes the emotional neglect of other children and the erosion of normal family functioning because of the primary caregiver’s unavoidable absence. A participant expressed the painful awareness of divided attention:

‘My other children are feeling neglected at home … I know it. I am always in the hospital with one child, so who is the mother to the others?’ (P9, 45 years, female, unemployed)

A participant described the overwhelming expansion of responsibility:

‘I am taking care of my sister’s children plus my own. My own crisis is multiplied. My family’s needs are a mountain I can’t climb.’ (P10, 28 years, female, unemployed)

#### Sub-theme 3.3: Relentless caregiving responsibility

Participants described the demands of caring for their children with hydrocephalus and indicated the strain of having to remain constantly present, which further leads to exhaustion and limited opportunities for rest or personal time. A participant stated:

‘Looking after the child all the time is strenuous…she is always in my hands … my body aches constantly. There is no one to hand him to, no rest. The physical toll is a deep exhaustion that medicine can’t cure.’ (P19, 28 years, female, unemployed)

While another participant said:

‘Taking care of him is strenuous. He needs too much attention and time. It’s a 24-hour job with no shifts, no weekends off. Your muscles hurt, your back hurt, and you know tomorrow will be the same.’ (P10, 28 years, female, unemployed)

#### Sub-theme 3.4: Social isolation and stigma

The results also showed a significant contraction in social life and personal autonomy, as caregivers reported the loss of friendships, withdrawal from community participation, and a feeling of being trapped in the caregiving role, often physically confined to the hospital or home. This experience was articulated through two sad narratives. Some participants said:

‘I am not able to have a social life. Friends stop calling because you always say no. Weddings, gatherings, they happen without you. You are physically stuck in this small world of care, watching life go on for everyone else.’ (P4, 45 years, female, unemployed)

‘I can’t go to work or anywhere. Life feels stuck. The hospital is my world. My dreams, my movements, my choices … are frozen. I am caring, but I am not living. I’m just … here.’ (P18, 28 years, female, unemployed)

The findings further illustrate how caregivers face social marginalisation through a dual process of withdrawal and stigmatisation. This involves both the caregiver’s necessary retreat from community life and the experience of being met with fear or avoidance by others because of the child’s visible condition.

This compounding dynamic is powerfully captured in the participants’ accounts. Participants stated that:

‘People at home and at the clinic are afraid of the child. They stare, they whisper, they keep their distance. The stigma isolates us more than the illness itself. You feel like you and your child are seen as something to be feared, not helped.’ (P5, 27 years, female, unemployed)

‘I’m not attending weddings because of the child. People don’t understand. They think you’re choosing not to come. Slowly, the invitations stop. The isolation isn’t forced by others; it’s built brick by brick by misunderstanding.’ (P2, 40 years, female, unemployed)

### Theme 4: Formal social protection availability

Caregivers highlighted limitations in formal support systems, which contributed to a reliance on informal and often unstable sources of assistance. In the absence of consistent institutional and social protection support, caregivers depended on household members and partners engaged in irregular work to sustain care. These constraints were further reflected in limited engagement with available psychosocial services, indicating challenges not only in access but also in the acceptability and uptake of formal support.

#### Sub-theme 4.1: Reliance on informal support networks

The study participants described a heavy reliance on informal support structures that were inherently precarious. Their dependency was often placed on family assistance or irregular income from informal labour, resources characterised by their inconsistency and instability. As a participant explained:

‘I am supported by my husband with piece jobs. That’s our safety net – informal, unstable work. If that work dries up, we fall. Our entire survival depends on a fragile network of favours and day labour.’ (P10, 28 years, female, unemployed)

This vulnerability was further illustrated by another participant, who highlighted how informal care arrangements themselves can become a source of strain:

‘I left three kids at home alone. My informal network, my children looking after each other, is all I have. It’s not support; it’s a desperate compromise. The network is so fragile, held together by the oldest child being a parent too soon.’ (P14, 26 years, female, unemployed)

#### Sub-theme 4.2: Insufficient formal support

The study’s findings point to a fundamental and systemic deficiency in the support ecosystem surrounding caregivers. This deficiency is not a singular issue but a pervasive failure across both structured, professional channels and the organic, personal networks that families typically rely on, leaving caregivers in a state of unsustainable vulnerability. This was confirmed by a participant who said:

‘Not having money to go to hospital is the biggest problem. There is no help for that. They tell you to come for check-ups but don’t tell you how to get there when you have nothing. The support system stops at the hospital door.’ (P13, 25 years, female, unemployed)

Another participant expresses the same sentiment by saying:

‘I do not get a grant for the child. I use my own money to go up and down. The formal support you hear about doesn’t reach everyone. You are left to figure it out alone, with empty hands.’ (P17, 23 years, female, unemployed)

#### Sub-theme 4.3: Reluctance to access psychosocial services

The findings indicate that caregivers recognise severe psychological distress as a direct consequence of their circumstances, yet they view psychological intervention alone as insufficient to resolve their core challenges. This was supported by a participant who said:

‘I am not emotionally ok, but I don’t want to talk to a psychologist. Talking won’t fix the shunt. It won’t pay for transport. My problems are real and practical; adding more talk feels like adding another burden, not a solution.’ (P8, 25 years, female, unemployed)

Similarly, another participant said:

‘I’m afraid of the condition, worried about the future. But a psychologist? That feels like a luxury for a different kind of problem. My stress has a name and a medical file; I don’t see how talking changes that reality.’ (P20, 28 years, female, admin clerk)

## Discussion

This study provides a concerning account of the multidimensional burden experienced by caregivers of children with hydrocephalus in two South African teaching academic hospitals. Consistent with existing literature, caregivers in this study were predominantly young, unemployed mothers with limited educational attainment, reflecting broader structural vulnerabilities in caregiving contexts within LMICs (Aukrust et al. 2025; Bakare 2020; Barnes et al. 2023; Enslin et al. 2021). These intersecting social determinants of health, including poverty, significantly shape caregiving experiences and constrain caregivers’ ability to provide optimal care.

The findings reveal a complex interplay of structural, economic, and psychosocial challenges that are deeply interconnected. Caregivers described fragmented care pathways, repeated hospitalisations, and the ongoing need to navigate complex health systems. These experiences align with prior studies demonstrating that hydrocephalus management, particularly because of shunt complications such as infections and revisions requires frequent hospital admissions and prolonged caregiving engagement (De Souza et al. 2025; Mansoor et al. 2021). The recurrence of illness episodes exacerbates caregiver strain and contributes to long-term uncertainty.

A critical finding of this study is the inadequate provision of caregiver education and preparation. Participants reported receiving insufficient or inconsistent information regarding their child’s condition and care needs, which heightened anxiety and reduced confidence in caregiving. This is consistent with evidence suggesting that effective caregiver education is central to improving caregiving competence and reducing psychological distress (Keklik, Nazik & Çelebioğlu 2025). Conversely, the absence of structured, accessible educational interventions in under-resourced public health systems limits caregivers’ ability to manage complex conditions and exacerbates reliance on already strained healthcare services. While educational interventions have shown promise in LMIC contexts, their integration into routine care remains inconsistent because of workforce and resource constraints (Kantawala et al. 2023; Shahali et al. 2024).

Financial hardship emerged as a pervasive and compounding burden, particularly regarding transport costs associated with repeated hospital visits. Delays in receiving a surgical intervention often resulted in prolonged hospital stays, contributing to loss of income and employment instability. These findings are strongly supported by LMIC literature, which identifies out-of-pocket healthcare costs and limited social protection systems as key drivers of caregiver burden (Henry et al. 2023; Kisangala et al. 2024). In this study, financial strain extended beyond direct medical costs to include indirect costs such as lost employment and disrupted livelihoods, ultimately entrenching families in cycles of poverty. Similar barriers have been documented in other sub-Saharan African contexts, such as Malawi, where transportation challenges significantly impede access to surgical care (Varela et al. 2019). These findings highlight how structural inequities, together with the centralisation of specialised healthcare services, place a greater burden on socioeconomically vulnerable populations by creating additional barriers to accessing timely care.

Emotional and psychological distress was a prominent feature of caregivers’ experiences. Participants reported symptoms consistent with depression, chronic anxiety, fear, and social isolation. These findings align with established evidence demonstrating that caregiving in chronic neurological conditions is associated with increased risk of psychological morbidity (Knerlich-Lukoschus et al. 2024; Zahl et al. 2024; Zimmerman et al. 2021). The persistent uncertainty associated with hydrocephalus, coupled with limited support systems, contributes to heightened emotional strain.

The physical demands of caregiving were also substantial. Caregivers described continuous caregiving responsibilities, including lifting, monitoring, and constant supervision, often without respite. This aligns with caregiving literature, which identifies physical strain, fatigue, and long-term health consequences as common outcomes of sustained caregiving (Shahali et al. 2024). While physical burden is well documented in adult caregiving populations, it remains underexplored in paediatric neurological conditions, underscoring the contribution of this study to the field.

Family and social dynamics were significantly affected by caregiving responsibilities. Participants expressed feelings of guilt related to neglecting other children and disruptions in family roles. These findings are consistent with evidence showing that caregiving can negatively impact family functioning, sibling wellbeing, and interpersonal relationships (Geuze & Goossensen 2019). In addition, limited social support and weak community-based services further intensified caregiver burden. Studies in similar contexts have highlighted that caregiving often occurs in environments where formal support systems are minimal, leaving families to absorb the full responsibility of care (Mwiinga et al. 2023; Torres & Ohajunwa 2025).

Importantly, while some literature highlights resilience and adaptive coping among caregivers of children with chronic conditions, such narratives were less prominent in this study. This may reflect the compounded effects of economic hardship, inadequate healthcare support, and persistent uncertainty, which constrain opportunities for positive coping and adaptation. The findings therefore suggest that caregiver distress is not solely a consequence of the child’s condition but is significantly shaped by systemic and structural inadequacies.

Overall, this study contributes to the growing body of evidence on caregiver burden in LMICs by demonstrating how health system limitations, socioeconomic vulnerability, and insufficient support mechanisms converge to shape the lived experiences of caregivers of children with hydrocephalus.

### Limitations

The study captured caregivers’ experiences during hospitalisation at only two selected hospitals … in [and] provinces. Thus, the findings do not fully capture the challenges faced by caregivers at other tertiary institutions different provinces. Although the interviews were conducted by experienced physiotherapists who were briefed to maintain neutrality and use a standardised semi-structured interview guide, the possibility of interviewer influence and social desirability bias cannot be completely excluded, as participants may have provided responses they perceived as favourable or acceptable.

## Conclusion

This study highlights the profound and multidimensional burden experienced by caregivers of children with hydrocephalus in two teaching academic hospitals in SA. Caregiving in this context is shaped not only by the clinical demands of the condition but also by structural limitations in the health system, financial hardship, and insufficient psychosocial support. These intersecting challenges place caregivers at significant risk of physical, emotional, and economic strain. Addressing these burdens requires a holistic approach that integrates caregiver education, financial support mechanisms, and strengthened health system responsiveness. Interventions that recognise and support caregivers as central partners in the healthcare of their children are essential to improving both caregiver wellbeing and child health outcomes in resource-constrained settings.
